# Cochlear Inflammaging in Relation to Ion Channels and Mitochondrial Functions

**DOI:** 10.3390/cells10102761

**Published:** 2021-10-15

**Authors:** Parveen Bazard, Jennifer Pineros, Robert D. Frisina, Mark A. Bauer, Alejandro A. Acosta, Lauren R. Paganella, Dominika Borakiewicz, Mark Thivierge, Freyda L. Mannering, Xiaoxia Zhu, Bo Ding

**Affiliations:** 1 Department of Medical Engineering, College of Engineering, University of South Florida, Tampa, FL 33620, USA; parveen1@usf.edu (P.B.); pinerosj@usf.edu (J.P.); mabauer@usf.edu (M.A.B.); aaacosta@usf.edu (A.A.A.); lpaganella@usf.edu (L.R.P.); borakiewicz@usf.edu (D.B.); markt2@usf.edu (M.T.); xiaoxiazhu@usf.edu (X.Z.); ding1@usf.edu (B.D.); 2 Global Center for Hearing and Speech Research, University of South Florida, Tampa, FL 33612, USA; fmanneri@iu.edu; 3 Department Communication Sciences and Disorders, College of Behavioral & Communication Sciences, Tampa, FL 33620, USA; 4 Morsani College of Medicine, University of South Florida, Tampa, FL 33620, USA

**Keywords:** aging, inflammaging, auditory system, age-related hearing loss, cochlea

## Abstract

The slow accumulation of inflammatory biomarker levels in the body—also known as inflammaging—has been linked to a myriad of age-related diseases. Some of these include neurodegenerative conditions such as Parkinson’s disease, obesity, type II diabetes, cardiovascular disease, and many others. Though a direct correlation has not been established, research connecting age-related hearing loss (ARHL)—the number one communication disorder and one of the most prevalent neurodegenerative diseases of our aged population—and inflammaging has gained interest. Research, thus far, has found that inflammatory markers, such as IL-6 and white blood cells, are associated with ARHL in humans and animals. Moreover, studies investigating ion channels and mitochondrial involvement have shown promising relationships between their functions and inflammaging in the cochlea. In this review, we summarize key findings in inflammaging within the auditory system, the involvement of ion channels and mitochondrial functions, and lastly discuss potential treatment options focusing on controlling inflammation as we age.

## 1. Introduction

Our population continues to grow older because of improved public health and medical care [1,2]. Specifically in the US, the number of people above 65 is projected to expand from 52 million in 2018 to 95 million in 2060, with the proportion of those 65 years and older increasing from 16% to 23% of our population [3]. Our increased aging population presents new medical challenges, due to age-related medical co-morbidities. Aging is a complex, slow, and deleterious process that happens at molecular, cellular, and tissue levels throughout our body. It weakens our immune system, and most body systems lose structural and functional integrity. Hence, we become susceptible to various age-related diseases, including cardiovascular and neurodegenerative disease, diabetes, various cancers, etc. [4,5,6,7]. There is no single underlying mechanism to explain these phenomena, but inflammation is tightly coupled with aging, and the association of inflammation plasma markers such as C-reactive protein, a clinical inflammation biomarker; interleukin-6 (IL-6); white blood cell counts (WBC); and other markers, with various aging diseases has been widely reported [8,9,10,11,12,13].

“Inflammaging” has a subtle definition, which is distinguished from “inflammation”, and is described as the low-grade, chronic, systemic inflammation in aging, in the absence of overt infection (“sterile” inflammation), and is a highly significant risk factor for both morbidity and mortality in the elderly [14,15,16]. Our immune system tries to fight off external threats such as infection due to injury, trauma, and other disorders, with an inflammatory response via complex interactions inside the affected tissue/body part. Normally, this leads to healing and the body goes back to homeostasis. As our immune system becomes older, it attains a higher inflammation state, chronic inflammation—the slow build-up of inflammation levels of the body: “inflammaging”, which can affect otherwise healthy body tissues and systems [17,18,19,20]. This can lead to diseases such as diabetes, hypertension, Parkinson’s, obesity, atherosclerosis, cardiovascular diseases, etc. [21,22,23,24,25,26,27,28,29,30,31,32]. Furthermore, apart from aging phenomena, genetic variations in genes related to inflammation also contribute to inflammaging and associated aging diseases [33,34,35]. In this review, we discuss the roles of chronic inflammation and the aging auditory system. We start with presenting age-related changes in the cochlea, relevant to chronic inflammation factors, followed by sections on the roles of ion channels/transporters and mitochondrial dysfunctions—two key parameters of the aged cochlea and cochlear inflammaging—and, finally, conclude with potential therapeutic strategies for combatting age-related hearing loss (ARHL).

## 2. Cochlear Aging

Presbycusis, also known as ARHL, affects over 10% of the world’s population, particularly those aged 65 and older [36,37,38,39]. It involves an irreversible, bilateral loss of auditory sensitivity that affects high frequencies first and progresses to lower frequencies with age. People who suffer from ARHL have reduced sensitivity to understanding speech in background noise, difficulty with sound localization and detection, and slowed central processing of acoustic information [40,41,42,43]. The causes and development of this disorder are multifactorial, and variable from individual to individual. However, research analyzing changes in the inner ear has provided key information to better understand ARHL and identify potential treatments to slow the progression of this disorder.

Changes in the aging cochlea—such as structural deterioration and functional losses—have been found to contribute to the development of presbycusis. A myriad of anatomical changes are involved in ARHL. A commonly referenced framework credited to Schuknecht describes the degradation of these structures in relation to ARHL [44,45,46,47]. According to this framework, presbycusis can be categorized into four groups: sensory, neural, strial, and cochlear conductive [47]. Major regions such as the stria vascularis (SV), organ of Corti, and cochlear neurons are key components of this age-related pathology. Schuknecht and Gacek [47] also include degeneration of the spiral limbus and spiral ligament in their framework. The progression of presbycusis in these regions could happen independently from each other or combined. They also noted that about 25% of ARHL cases do not fit neatly into the four groups; hence, they referred to these cases as indeterminate presbycusis.

Strial, or metabolic, presbycusis is the degradation of the SV. The SV is responsible for the generation of the endocochlear potential (EP) and maintaining the high potassium ion (K^+^) content of the endolymph [48,49,50,51]. This is key for the normal signal transduction of sound information into the electrochemical responses of hair cells (HCs), which synapse on auditory nerve fibers to carry sound information to the brain. The stria may degrade up to 30–40% before any changes in audiograms are seen. Degradation of the SV normally occurs in the mid-cochlear to apical regions, and is hypothesized to be related to downregulation of the EP and depletion of Na,K-ATPase, sodium potassium chloride cotransporter isoform 1 (NKCC1), and other ion channels/proteins in the cochlear supporting cells, including the lateral wall [38,52,53,54,55,56,57,58,59,60,61,62]. For instance, Schulte and Schmiedt [60] found that EP levels decrease first in the apex of the cochlea before decreasing in other regions of the cochlea as aging occurs, which is correlated with the expression levels of Na,K-ATPase found in the stria. Na,K-ATPase is important for ion transport, and there is a decrease in its presence with age in the stria [63], where its expression was found to be reduced up to 80% in aged CBA/CaJ mice [48,53]. Moreover, localization of decreasing Na,K-ATPase expression was seen to occur in the same regions where EP levels dropped [63].

A growing area of research related to metabolic presbycusis is studying changes in the vasculature of the stria. Degenerative changes in capillaries, from the narrowing of vessel lumens, to complete degradation of the vessels in the stria, have been discovered in ARHL animal models [64,65,66,67]. In aged C57BL/6J mice, capillary density and vessel diameter were significantly reduced [65]. Temporal bones of humans with presbycusis showed that the vascular walls of the capillaries in the stria were thickening [68]. Some of these changes were found to happen more in the basal turn compared to the higher turns [64]. However, other animal studies (gerbils) showed a normal vasculature pattern in the middle and upper basal turns, while the rest of the stria showed capillary deterioration with age [67]. It is evident that more research is required to study the exact relationships between strial vascular degeneration and ARHL.

Elevations in hearing thresholds that are standard with ARHL stem from the degeneration of the organ of Corti, specifically the loss of cochlear outer hair cells (OHCs). This coincides with what Schuknecht called sensory presbycusis. Wu et al. [69] used cytocochleograms from different human age groups, middle to old age, to show the HC loss that occurs with age. The number of HCs from a young person were significantly greater than those seen in older subjects. Another characteristic of sensory presbycusis is the early loss of HCs at higher-frequency cochlear regions (basal part of the cochlea); thus, explaining hearing loss in the high-frequency range first, followed by hearing loss in the low-frequency range. It is also reported in the Schuknecht classifications that sensory presbycusis accounts for at least 5% of all ARHL cases [70].

In the past few years, a new mechanism, involving the loss of key synaptic features connecting inner hair cells (IHCs) with the dendrites of auditory nerve fibers, emerged, called “cochlear synaptopathy”. This mechanism causes the decline in cochlear neuronal synapses, which are vulnerable aspects of auditory aging, while HCs are preserved [38,71,72,73]. Studies utilizing CBA/CaJ mouse models have identified these ribbon synapses and found that they diminished with age, while HC counts remained normal [71,72]. Kujawa and Liberman discovered that after being exposed to loud noise—that would not cause permanent threshold shifts—young adult CBA/CaJ mice developed accelerated ARHL and irregular loss of synapses with aging [71,74]. Further animal studies from Parthasarathy and Kujawa [72] showed more evidence of synapses disappearing with age as HC expression remained nearly constant.

## 3. Cochlear Inflammaging

Though explicit mechanisms involving chronic inflammation and the aging auditory system have yet to be established, recently, they have garnered attention. For instance, Verschuur and coworkers [75] studied the association of inflammation biomarkers and ARHL in humans, by looking at cross-sectional data on audiogram and inflammatory markers from 343 males and 269 females aged between 63–74 years—Hertfordshire, UK population sample. Four inflammatory markers, white blood cell count (WBC), neutrophil count, interleukin-6 (IL-6), and C-reactive proteins, were positively associated with hearing thresholds in older people, though no association was observed with maximum audiometric slope. The analysis identified was adjusted for older age, smoking, noise exposure history, and male gender, as factors associated with higher hearing thresholds. To confirm the findings further, Verschuur et al. [76] conducted the study with another population cohort, examining data from the Medical Research Council national study of hearing, with data from four locations in the UK. WBC was used as the marker of inflammaging. A significant correlation was observed between WBC and the pure-tone average threshold for ages 60 and above. Additionally, the association became stronger with age. Lassale et al. [77] also found an association with WBC and hearing impairment in older adults in a longitudinal study (4789 participants, aged 50–93—median age 63 years at the baseline), though no correlation was observed with two other inflammation makers—C-reactive protein and fibrinogen. Sardone et al. [78] reported that pro-inflammatory foods with high sugar contents, such as beer and liquor, were associated with ARHL, but anti-inflammatory foods such as fruits, vegetables, nuts, and wine were not related to hearing in a cross-sectional study conducted in 734 participants. These human studies strongly indicate relationships between inflammation and the aging cochlea.

**Figure 1:**
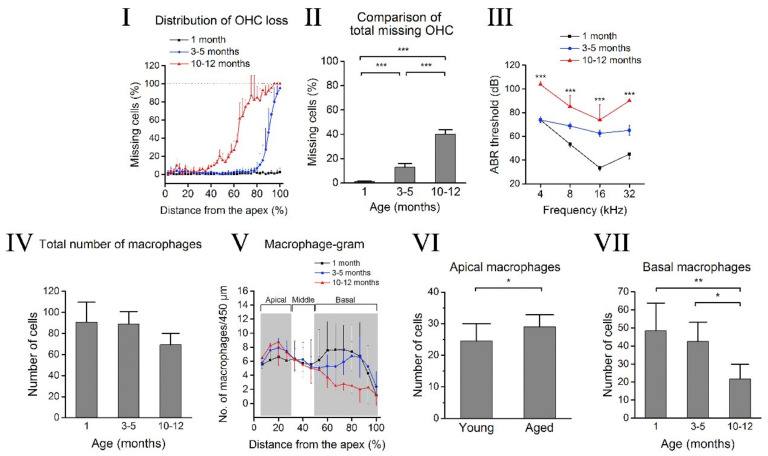
Age-related inflammation changes in the C57BL/6J mouse cochlea. (**I**) Cochleogram showing progressive OHC loss throughout the basilar membrane for different age groups of mice: young (*n* = 13), intermediate-aged (*n* = 20), and old (*n* = 4) mice. Note that OHC loss starts from the cochlear basal end. (**II**) The number of missing OHCs is significantly higher in older age groups compared to the younger groups (*p* < 0.0001). (**III**) ABR thresholds at 4 different frequencies show significantly higher thresholds for the older animal groups compared to the younger, baseline animals (*p* < 0.001). (**IV**) Total number of macrophages in the sensory epithelium decreases between three age groups: 1 month (*n* = 11), 3–5 months (*n* = 16), and 10–12 months (*n* = 4). (**V**) Macrophage-gram shows the number of macrophages in the basal region decreases with age at a higher rate compared to the apical and medial regions. (**VI**) The difference in microphage amount in the apex is significantly higher in aged animals (3–12 months) compared to the young animals (1 month) (*p* < 0.05). (**VII**) A significant reduction in basal macrophages is observed between the age groups (*** *p* < 0.001, ** *p* = 0.002, * *p* = 0.014). From Frye et al. [82], with permission from the publisher.

Traditionally, the cochlea, since it has a tight intrastrial fluid–blood barrier like the blood–brain barrier, was considered as an immune-privileged organ. However, since the first confirmation of various immune cells such as lymphocytes, monocytes, plasma cells, and macrophages in the guinea pig’s endolymphatic sac, by Rask-Andersen and Stahle [79], there have been several reports confirming the presence of immune system markers in cochlear fluids, such as macrophages. For instance, Zhang and co-workers reported a layer of perivascular-resident macrophages such as melanocytes (PVM/Ms) involved in the maintenance of the intrastrial fluid–blood barrier integrity, which are sandwiched between the marginal and basal cells of the SV [80,81]. Frye et al. [82] studied aging effects on basilar membrane macrophages in C57BL/6J, a well-known mouse model for ARHL studies. Three different groups of animals were used for the study: 4–6 weeks, 3–5 months, and 10–12 months. Progressive ARHL was observed, starting with high-frequency hearing loss in the basal region and moving to the apex region with age. Immunostaining was used to identify two inflammation markers: CD45 protein, a leukocyte marker, and F4/80, a macrophage-specific marker. Morphology changes were observed for both the 3–5 months and 10–12 months groups as compared to the young adult group—4–6 weeks old, especially in basal and middle cochlear turns. Enlarged and irregular-shaped cell bodies with a grainy appearance were observed in the basal region. Additionally, basal sections exhibited more prominent declines in the total number of macrophages with aging compared to the apical region. Another interesting finding was that macrophage activity was enhanced in cochleae with a higher degeneration of OHCs as compared to cochleae with less severe OHC lesions, and macrophage activity was reduced once cells were degenerated along with elevation in ABR thresholds, as depicted in Figure 1. Shi et al. [83] discovered increased levels of various inflammation markers; interleukin-6 (IL-6), interleukin-1β (IL-1β), tumor necrosis factor-α (TNF-α), and Nod-like receptor 3 (NLRP3) in the aging mouse cochlea. Lastly, Menardo et al. [84] provided evidence about the involvement of oxidative stress, chronic inflammation and apoptosis, as key mechanisms of ARHL in the senescence-accelerated mouse prone 8 (SAMP8) strain. Additionally, whole transcriptome analysis of C57BL/6 cochleae revealed that 731 genes were differentially expressed (379 upregulated and 352 downregulated) in the cochlea of aging mice at 12 months, as compared to the young adult mouse cochlea at 4 weeks old, and genes associated with inflammation were found to be significantly modulated [85].

Apart from direct evidence of inflammation involvement in ARHL, there are studies demonstrating that inflammation plays a key role in other auditory pathologies such as noise-induced hearing loss and ototoxicity that have many mechanistic similarities with ARHL, though on a more acute time scale. The cochlear inflammation cytokines IL-6, TNF-α, and IL-1β increase due to noise exposure [16,86,87,88,89,90]. For instance, Nakamoto et al. [91] exposed CBA/N male mice to a 130 dB sound with a center frequency of 4 kHz for 3 h. There was an increase in inflammatory cytokines IL-6 and IL-1β gene expression, and administration of 0.5% geranylgeranyl acetone in chow feed decreased these biomarkers. Similarly, Zhang et al. [92] exposed Sprague Dawley rats to 120 dB SPL white noise for 8 h/day for 2 consecutive days and found that the Toll-like receptor-4 (TLR-4) signaling pathway was present for cochlear inflammation after the exposure. There are other reports along similar lines, confirming the roles of inflammation after noise exposure [93,94,95,96,97]. These reports support the idea that inflammation is a risk factor for cochlear chronic pathologies such as ARHL.

Chronic inflammation mechanisms are complex, and many inter-related processes contribute to them, e.g., redox-stress-driven alterations at cellular and molecular levels, mitochondrial damage, oxidative stress, hormonal imbalances with age, epigenetic changes, etc. [6,7,8,98,99,100,101]. Ion channel imbalances and mitochondrial dysfunction are considered as two key events in the aging cochlea. In the next sections, we will discuss how these two parameters affects cochlear inflammaging.

## 4. Cochlear Inflammaging and Ion Channels/Transporters

**Figure 2:**
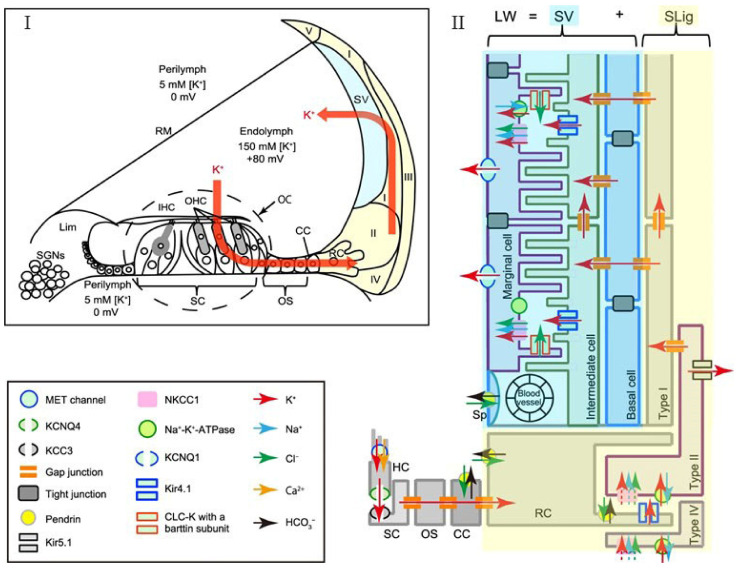
Diagram depicting Na,K-ATPAse, NKCC1 ion transporters, and K^+^ channels involved in ion circulation in the cochlear lateral wall. (**I**) Recirculation of K^+^ between the HCs into the endolymph of the scala media during auditory transduction. The ion channels involved in K^+^ recycling play a vital role in generating the EP and maintaining the lymphatic homeostasis needed for normal hearing. (**II**) NKCC1, Na,K-ATPase, KCNQ1, KCNQ4, Kir 4.1, Kir 5.1, and KCC3 are some various ion transporter channels that play a role in K^+^ circulation and endocochlear potential generation in the SV. HC: hair cells; IHC: inner hair cells; OHC: outer hair cells; OC: organ of Corti; SC: supporting cells; OS: outer sulcus cells; CC: Claudius cells; RC: root cells; Lim: spiral limbus; RM: Reissner’s membrane; SLig: spiral ligament; Sp: spindle cell. From Watabe et al. [108] (Figure 1), with permission from the publisher.

**Figure 3:**
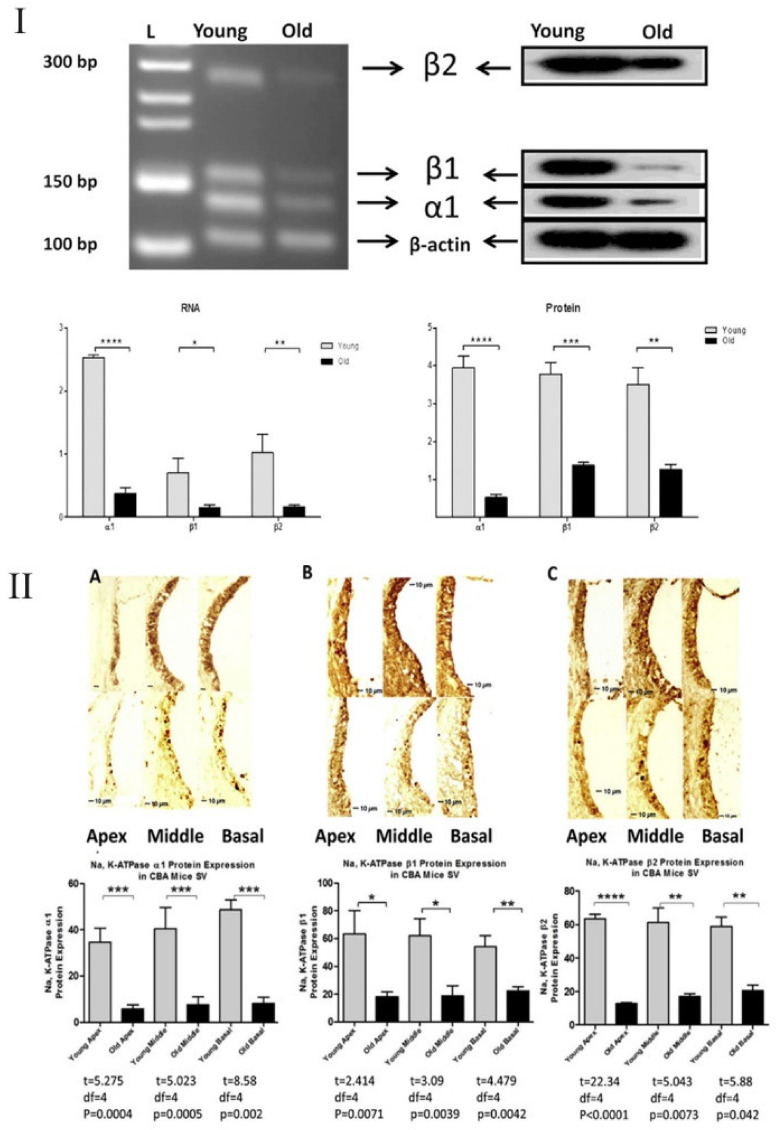
Na,K-ATPase subunits expression decreases in aging CBA/CaJ mouse cochlea. (**I**) α1, β1, β2 subunits of Na,K-ATPase at both protein and gene expression levels significantly decreased between young adult (3 months) and old (30 months) CBA/CaJ mice. (**II**) Immunohistochemistry performed on cochlear cross sections confirm the decrease in α1, β1, β2 subunits of Na,K-ATPase expression levels. * *p* < 0.05, ** *p* < 0.01, *** *p* < 0.005, **** *p* < 0.001. From Ding et al. [53], with permission from the publisher.

Correlations between the functionality of ion channels and inflammation have been a research topic for some organ systems. It was reported that disruption in ion channel transport is associated with inflammation [102,103,104,105]. Relevant to the present article, Na,K-ATPase is a sodium–potassium cation pump found in the plasma membrane of most mammalian cells, including inner ear cells. Transporting ions against the concentration gradient, this enzyme complex helps to maintain low Na^+^ and high K^+^ intracellular concentrations [106]. Two subunits comprise Na,K-ATPase: the α subunit, which has four isomers, and the β subunit, which has three isomers. While expressed in SV, a specialized organ in the lateral wall of the cochlea, and other cochlear cells, Na,K-ATPase helps to generate and maintain the EP at high positive values ~80–100 mV [107] and is a key cochlear ion transporter. Figure 2 shows the ion transporters and ion channels in the cochlea and its lateral wall. Na,K-ATPase also serves as a specific binding site to cardiotonic steroids such as ouabain and exhibits non-specific binding for reactive oxygen species (ROS) [108,109]. There are reports indicating that pathways such as NKCC1 and Na,K-ATPase are closely tied to EP disruption, for example, during presbycusis. Figure 3 shows the aging decline of Na,K-ATPase in the CBA/CaJ mouse model. NKCC1 is another key channel in the inner ear and it declines with aging. [48,110,111,112]. Lui and coworkers showed progressive aging declines in NKCC1 protein and gene expressions in C57BL/6J mice [112]. Ding and colleagues further confirmed this finding in CBA/CaJ mice, another well-known animal model for carrying out aging auditory studies [111,113]. Diaz et al. [114] generated NKCC1^+/−^, α_1_-Na,K-ATPase^+/−^, and α_2_-Na,K-ATPase^+/−^ single-heterozygous mutant mice as well as double-heterozygous mutants NKCC1^+/−^ α_1_^+/−^ and NKCC1^+/−^ α_2_^+/−^, and performed ABRs and DPOAEs, as well as recording EPs through the insertion of a microelectrode into the SV of scala media. In this study, the three single-heterozygous mutants demonstrated various degrees of ARHL loss, and the double NKCC1^+/−^ α_1_^+/−^ mice exhibited severe ARHL later (51–60 weeks) compared to the single-heterozygous counterparts (31–40 weeks). This hearing loss was exhibited without substantial deterioration of the cochlea and HCs, indicating that the ion channel expression declines were most likely responsible for the observed presbycusis [114].

Although inflammation and Na,K-ATPase, NKCC1 relationships have not been well studied in the cochlea, some important clues can be gleaned from a closer look at the effects of inflammation on Na,K-ATPase ion channel expression. Schmidt et al. [115] investigated the regulation of renal sodium transporters during severe inflammation both in vivo and in vitro. For in vivo study, high-dose injections of lipopolysaccharide (LPS) were administered to induce inflammatory responses, as LPS has been shown to increase the production and release of proinflammatory cytokines [116]. LPS injections in mice resulted in a strong decrease in Na/H exchanger and Na,K-ATPase expression. For in vitro testing, cytokines were shown to reduce the expression of outer medullary K^+^ channels, epithelial sodium channels, and Na,K-ATPase in cortical collecting duct cells [115]. Similarly, Allgayer et al. [117] explored the expression of Na,K-ATPase in patients with inflammatory bowel disease (IBD). A total of 19 patients who suffered from IBD of mild-to-moderate disease activity (15 of which suffered from ulcerative colitis, while the other 4 suffered from Crohn’s colitis) were selected for enzyme assays. These studies not only demonstrated that Na,K-ATPase activity was reduced due to inflammation, but it also showcased a statistically significant negative correlation between the degree of inflammation and the expression of this ion channel [117]. There are various other studies linking Na,K-ATPase dysfunction or alterations with inflammaging symptoms. In one study, a high-salt diet was administered to Dahl salt-sensitive rats after disturbing the Na,K-ATPase signaling pathways in the renal proximal tubule [118]. The rats’ higher urine Na^+^ concentrations compared with those of the control group indicated that Na,K-ATPase did not efficiently resorb Na^+^ ions. The research also indicated that impairing the Na,K-ATPase pathways resulted in salt-induced hypertension in the rats. Since hypertension is also a result of oxidative stress, which contributes to inflammaging, we would expect similar hypertensive or inflammaging effects with Na,K-ATPase impairment in cochlear cells. Sodhi et al. [119] also described the Na,K-ATPase signaling pathway, called the Na,K-ATPase oxidant amplification loop (NKAL), that produces ROS, which can exacerbate inflammaging effects. In summary, ROS, when binding non-specifically to Na,K-ATPase, creates a signaling cascade from the ion channel, to Src—a kinase protein playing a role in cellular processes such as growth, proliferation, differentiation and physiology—to epidermal growth factor receptor (EGFR), which generates more ROS [120]. It was observed that by inhibiting the NKAL, they could lessen traits of certain diseases, including uremic cardiomyopathy and obesity [120,121]. Qu et al. [122] analyzed the potential for using 3S, 3’S-ASTaxanthin’s high antioxidant and anti-inflammation abilities to reduce NKAL activity from reperfusion, which is performed to treat myocardial infarction damage, yet can also cause high ROS generation and subsequent inflammation [122]. These researchers found that cell injury detection was significantly lower in 3S, 3’S-AST-treated cardiomyocytes compared with those treated with H_2_O_2_. 3S, 3’S-AST was also found to inhibit ROS-induced cardiomyocyte apoptosis and reduced phosphorylation of Src, thus attenuating the NKAL.

Na,K-ATPase expression has also been linked to the generation of ROS through cardiotonic steroid (CTS)-mediated signal transduction [123]. The Xie model for Na,K-ATPase signaling proposes a dynamic in which the Na,K-ATPase α1 subunit operates as a negative regulator of Src kinase, by inactivating the species through binding. However, conformational shifts brought about by CTS binding or oxidation can cause bound Src kinase to become active. The active Src kinase then transactivates EGFR and begins a signal cascade which increases ROS abundance [123]. The Xie model is one proposed explanation for the observed interactions between Na,K-ATPase and Src kinase. Yet, the signaling pathway which correlates Src activation and increases in ROS has been well documented, and labeled the Ras–Raf–MEK–ERK pathway [124]. Initially, a Ras protein becomes activated by EGFR. This activation, in turn, results in mitogen-activated protein kinase (MAPK) protein activation as well as an increase in calcium ion concentrations, leading to the opening of mitochondrial ATP-sensitive K^+^ channels in addition to increasing the mitochondrial generation of ROS [125,126]. These interactions between Na,K-ATPase and ROS are of particular interest, since they are key signaling molecules in the development and progression of numerous inflammatory disorders [127].

Similarly, there are reports confirming the involvement of NKCC1 in chronic inflammation in various body parts [128,129,130]. NKCC1 regulation has been experimentally correlated with inflammation through nuclear factor kappa B (NF-κB) signaling pathways. Gong et al. [131] studied the role of NKCC1 in inducing inflammation in cases of surgical brain injury (SBI). Additionally, the study sought to determine whether NKCC1 controls the release of IL-1β, IL-6, and TNF-α via the phosphorylation of NF-κB in microglia. In order to test this, the study used male Sprague Dawley rats as animal models. These were then randomly assigned into four groups and injected with either a sham or a variant of SBI; relevant protein, microglia, and cytokine expressions were then recorded. It was determined that SBI-induced increases in p-NKCC1 correlate with exacerbated neuroinflammation, brain edema, and nerve function injury. Furthermore, bumetanide (BUM)-mediated inhibition of NKCC1 was found to reduce the phosphorylation of the NF-κB signaling pathway; thus, reducing the release of inflammatory factors IL-1β, IL-6, and TNF-α [131]. BUM is a loop diuretic that is known to inhibit NKCC1 and NKCC2. However, further research must be conducted to characterize its relationship to inflammation and the NF-κB signaling pathway. Lastly, other studies have also linked NKCC1 inhibition to reduced fluid clearance in inflammatory disorders such as ischemia–reperfusion [132] and goblet cell mucus secretion defects [130]. Further work is needed to establish these ion channel links in the cochlea and other auditory areas.

Apart from ion transporters such as Na,K-ATPase and NKCC1, K^+^ channels are involved in cochlear ion transport [48,50,62,133,134,135,136]. Amongst the more than 100 potassium channels which have been identified, three inner ear K^+^ channels—inwardly rectifying K^+^ channels (Kir), voltage-activated K^+^ channels (Kv), and calcium-activated K^+^ channels (BKCa)—have been linked to inflammatory processes [137,138]. Of particular interest are Kir channels, involved in K^+^ circulation in the cochlea. A study by Xie et al. [139] determined that Kir ion channels are highly susceptible and regulated by pH—a significant parameter affected by the activation of immune inflammatory cells [139,140,141,142].

Although further research directly linking K^+^ ion channel functionality and inflammaging is still needed, current findings show some promise in correlating inflammation with ion channel activation. Further, the Kir and Na,K-ATPase channels (whose activation has already been correlated with inflammation in renal and musculoskeletal systems) are of importance in the physiology of the auditory system. By participating in the regulation of the EP, intracellular volume, and K^+^ circulations, these ion channels are integral for the normal functioning of the inner ear. Lastly, Franceschi and Campisi’s studies on inflammaging suggest that the chronic inflammation of aging is responsible for the low-grade production of pro-inflammatory cytokines, including IL-6, IL-1β, and TNFα [18,143]. These cytokines appear to be significant mediators of age-related damage to a wide variety of tissues which is an encouraging link hinting at the non-localized mechanisms that underlie inflammation-mediated ion channel activation [115].

## 5. Necroptosis, Mitochondrial Dysfunction, and Inflammaging in the Inner Ear

Recent research has begun to reveal the interconnected influences observed in aged systems which exhibit chronic inflammation, or inflammaging, along with mitochondrial and ion channel dysfunction. Interestingly, these interactions appear to be cyclical in nature, forming a positive feedback loop, where dysfunction leads to inflammation which leads to more dysfunction, and so on [144]. More importantly, in the field of hearing science, investigating these relations observed in other systems could allow for novel therapeutic targets.

As previously described, inflammaging is a chronic, low-grade inflammation and is the result of degraded “damage signal” receptors, with a corollary activation of the innate immune system [144,145]. Inflammaging has been shown to negatively affect mitochondrial processes, resulting in further negative downstream effects. This has been seen in neurodegenerative disorders, where inflammatory mediators alter mitochondrial metabolism through microglial activation [146]. The well-known cytokine TNF-α, especially, plays a large role as it has been shown to impede the mitochondrial oxidative phosphorylation needed for ATP production as well as impair the electron transport chain (ETC) complexes both in vitro and in vivo [147,148]. In inhibiting ETC complexes, ATP production is also reduced and upregulates reactive oxygen species (ROS), responsible for accumulated mtDNA mutations [149,150,151,152,153,154,155]. Accumulated mtDNA mutations may also lead to inefficient polypeptide production in the ETC, causing further oxidative stress and damage [154,156,157]. Additionally, current research suggests that an imbalance between mitophagy and mitochondrial biogenesis are also related to human pathologies and aging [158]. Effective mitophagy, as well as autophagy, is critical for cellular homeostasis and efficient bioenergetic maintenance, where inflammation has been shown to inhibit these processes [152].

**Figure 4:**
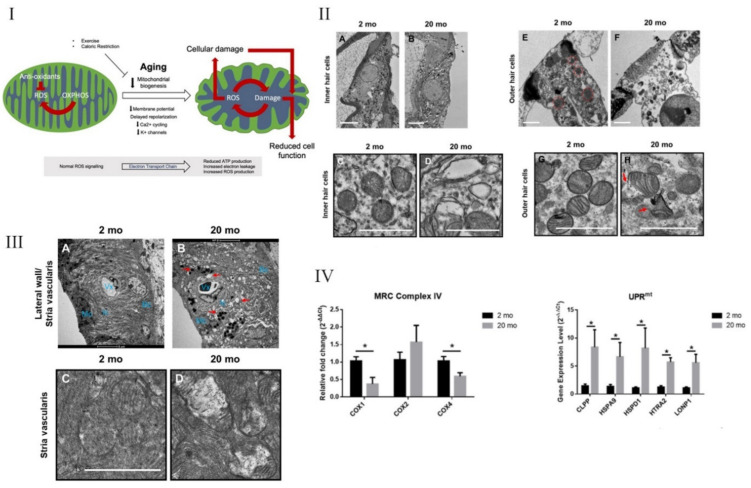
Mitochondrial changes within the aging cochlea. (**I**) Schematic drawing illustrating the production of ROS through regular oxidative (OXPHOS) activity. ROS production is normally kept under control via anti-oxidant systems; however, during aging, mitochondrial dysfunction accumulates and reduces ROS control. The resulting increase in ROS production causes cellular and further mitochondrial damage, leading to apoptosis. (**II**) (**A**) Young IHCs with normal appearing mitochondria with clear, distinct lamellar cristae (**C**). (**B**) Older IHCs with damaged mitochondria that have (**D**) missing or damaged cristae. (**E**) Young OHCs, also with (**G**) clear, well-defined lamellar cristae. (**F**) Older OHCs show aging effects on the mitochondria, such as swollen cells and chromatin compaction. (**H**) The cristae from the OHCs are distorted and show a broken wall. Scale of (**A**,**B**) is 5 µm and (**C**–**H**) is 1 µm. (**III**) (**A**) Transmission electron microscopy (TEM) images of young SV shows normal expression of marginal (mC), intermediate (Ic), and basal (Bc) cells, and the blood vessels (Vx). (**B**) An aged cochlea shows a degraded SV degeneration. This aging is depicted by an increase in vacuoles and pigmentation (red arrows), along with enlarged intercellular spaces. Scale for (**A**,**B**) is 5 µm. (**C**) Young SV with normal mitochondria with well-defined cristae. (**D**) Aged SV showing marred mitochondria with altered cristae. Scale for (**C**,**D**) is 1 µm. (**IV**) Gene expression changes in mitochondrial respiratory chain (MRC) complex IV significantly decrease with aging, while unfolded protein response (UPR) gene expression levels between young and old animals significantly increased (* *p* < 0.05). From Lyu et al. [159] and Strickland et al. [162], with permission from the publisher.

Inflammaging has also been shown to result in the immune response, necroptosis. Here, intracellular molecules are released from dying cells, culminating in a programmed cell death process that exhibits necrosis-like morphological characteristics. These characteristics include permeabilized intra- and extracellular release of organelles and cellular contents into extracellular media, inducing inflammation [159]. Lyu et al. [159] compared young adult (2 months) C57BL/6J males to old (20 months) males of the same strain (Figure 4). In the C57BL/6J mouse strain, accelerated high-frequency hearing loss is observed within 3–6 months, with profound hearing loss by 15 months of age, and thus is a great model for investigating mitochondrial metabolism as well as cochlear degenerative pathways. In this study, it was observed that along with upregulated proinflammatory cytokines, i.e., IL-1β, IL-6, and TNF-α, in older mice, comparatively, there were also distinct morphological changes in the mitochondria, as well as the upregulation of both mitochondrial damage and necroptosis markers. Transmission electron microscopy (TEM) structural analysis revealed disorganized and damaged cristae of the mitochondria in older mice. Reverse transcription polymerase chain reaction (RT-PCR) analysis of aged cochlear tissue also revealed decreased cyclooxygenase 1 (COX1) and COX4 expression, indicating mitochondrial dysfunction, since COX1 and COX4 are important subunits in the ETC. In addition to these findings, TNF-α and its associated receptor are known to regulate necroptosis, as evidenced by increases in the receptor-interacting serine/threonine-protein kinase 1 and 3 (RIPK1, RIPK3) and pseudokinase mixed-lineage kinase domain-like (MLKL). Taken together, these results show that inflammaging, mitochondrial dysfunction, and necroptosis all occur within the aged cochlea [159]. In another study examining the relationship between mitochondrial dysfunction and hearing loss, C57BL/6J mice were also used to simulate sleep apnea using a chronic intermittent hypoxic (CIH) environment. Young (8 weeks) male mice were subjected to 4 weeks of this environment and analyzed for mitochondrial markers as well as hearing thresholds in comparison to control mice under normal oxygen conditions. The results for CIH mice again support the important role of cochlear mitochondria where they were misshapen with apparent structural damage and reduced in number within the IHCs. The functionality of the cochlea was also affected, as CIH mice exhibited increased hearing thresholds, indicating a decline in auditory function. Additionally, mtDNA copies were reduced while mitochondrial markers such as peroxisome proliferative activated receptor-γ (PPAR-γ), co-activator 1α (PGC1- α), and mitochondrial transcription factor A (Tfam) were increased. PGC1- α and Tfam are promoters of biogenesis and mtDNA transcription, respectively. It was hypothesized that these proteins were upregulated in response to the oxidative damage occurring to the mitochondria, to try to maintain cellular homeostasis, and morphological defects in peroxisomes have been associated with various aging pathologies [160,161].

Intriguingly, mitochondrial damage also appears to be an initiator of inflammation through the extracellular release of damage-associated molecular patterns (DAMPs). DAMPs are composed of mtDNA, N-formyl peptides created from mitochondrial-encoded proteins, and unique phospholipids found in the mitochondrial membrane which are released into the extracellular space. Circulating mtDNA and formyl peptide DAMPs have been observed, recruiting neutrophils and cytokines in response to cellular trauma. They may also activate the NLRP3 inflammasome, which effects a downstream activation of caspase-1 and subsequently the proteolytic activation of the proinflammatory cytokines IL-1β and IL-18 [151,152,162]. This caspase-1 activation can induce mitochondrial damage, through downregulation of the autophagy activators, phosphatase and tensin homolog–induced putative kinase 1, which also regulate mitophagy [163]. Reduced autophagy and mitophagy then allows for a buildup of damaged mitochondria and senescent cells in tissue where they should have been cleared away. Here, senescent cells, as a result of aging, have been shown to exhibit proinflammatory properties through increases in cytokines such as IL-6 and TNF-α [164,165,166]. Mitochondrially generated ROS may also participate in NLRP3 activation, although the precise mechanism is unknown [151,162,167]. Furthermore, NLRP3 gene mutations have been responsible for autosomal-dominant DFN34 hearing loss [168,169,170].

In evaluating the effect of inflammaging on mitochondrial performance, it also appears that ion channels are greatly affected through these interactions as well. However, this relationship seems to be cyclical, as ion channels regulate mitochondrial function through the influx of relevant ionic species into both the cytosol as well as the inner membrane space of the mitochondria [162]. Of note, decreases in ion channel function with concomitant changes in ionic gradients have been postulated to drive the age-related declines observed in physiological systems [171]. For example, aging neurons have been shown to experience decreased mitochondrial Ca^2+^ cycling due to impaired Ca^2+^ channel functioning, delaying neuron repolarization following action potential firing, while also reducing mitochondrial membrane potential [172]. Mitochondrial K^+^ channels have also been shown to be reduced in density with aging. These reductions result in an increased risk for myocardial infarction as well as reduced neuronal activity [173]. The flux of ions (Ca^2+^, K^+^, Na^+^, H^+^) across the mitochondrial membrane, and thus the mitochondrial membrane potential, is an important process needed to maintain the flow of electrons through the ETC. Any perturbation then negatively influences the production of ATP, which as previously described has the potential to disrupt downstream physiological processes [174]. More specifically, ion channel dysfunction in the auditory system has been shown to negatively impact hearing by mainly impacting the K^+^ channels that affect the generation of the EP needed for hair cell depolarization. Here, age-related hearing disorders are often linked to a decrease in EP and reduction in K^+^ channel functionality; reductions which while yet unconfirmed, can reasonably be attributed to inflammation and mitochondrial dysfunction [48].

As mentioned previously, mitochondria are known activators of the inflammatory response. However, research in various cell models has also begun to show not only the negative effects of inflammation upon ion channel function, but also the upstream effects of cellular ionic imbalance upon mitochondria. Dysregulated ionic flux leads to membrane damage, which activates the NLRP3 inflammasome through ROS disequilibrium and ATP release [162]. This cyclical relationship, where inflammation and ionic dysfunction damage the mitochondria and thus result in more inflammation and dysfunction, perpetuates itself; thus, contributing to many age-related disorders associated with inefficient ATP production. It is important to note that many of these findings have been discovered in bodily systems unrelated to hearing. However, the intracellular pathways and routes of adaptation are equally applicable to the auditory system and provide important targets for future therapeutic development.

## 6. Intervention Strategies to Prevent Damage/Treat the Aging Cochlea

Currently, there is no FDA-approved drug for hearing pathologies, including ARHL. Present interventions such as hearing aids and cochlear implants are focused on compensating for hearing loss to help improve daily communication, but the majority of hearing aid users are unsatisfied with the results [175,176,177,178,179,180]. Progression in understanding the relationships between inflammaging and ARHL have allowed for advancements in research on strategies to treat the aging cochlea. Studies analyzing the effects of anti-inflammatory drugs on presbycusis are relatively new to the area of hearing research; thus, not many studies have been carried out on this. However, the existing literature shows promising results. The utilization of the glucocorticoid dexamethasone (DEXA) has shown protective effects on HCs by activating the PI3K/Akt and NFκB signaling pathways and defending against TNFα apoptosis [181]. Following application of DEXA and TNFα to Sprague Dawley rat pups’ cochlear explants, DEXA was shown to have protective effects on the HCs. TNFα is an inflammation-related cytokine that can be found in the cochlea and is linked to apoptosis [87,182].

Another anti-inflammatory drug currently under investigation is aspirin. Prolonged low-dose treatment of salicylate (aspirin) was found to upregulate the expression of prestin, encoded by the gene *SLC26A5,* a transmembrane motor protein expressed in OHCs, via the cyclo-oxygenases-II (Cox-II) independent pathways affecting OHC functionality [183,184,185]. Of note, mutations in SLC26A5 are responsible for the autosomal recessive form of deafness (DFNB61). Clinical trials using aspirin are also ongoing. There is currently a three-year study named the ASPREE-HEARING trial that aims to determine if low-dose aspirin administrated to people 70 years old or older (*n* = 1262) helps reduce the progression of ARHL [186]. The rationale for this study is that previous studies have shown that aspirin treatment decreases inflammatory markers such as TNF-α, IL-6, and thromboxane B2 (TXB2) and provides protective effects after ototoxic noise exposure [187,188,189]. Moreover, aspirin has been suggested to be used as a pro-inflammatory resolution mediator [190]. At low doses (~81 mg), aspirin upregulates aspirin-triggered lipoxin A4 (ATLA4), which inhibits eicosanoid thromboxane, which is useful for preventing vascular diseases that involve inflammation [191].

The use of hormone treatments is another intervention approach that has been investigated in recent years. Hormone imbalance has been closely linked with chronic inflammation [101,192,193]. One hormone studied is thyroid hormone. Previous literature has demonstrated that the thyroid is essential for normal cochlea development and may be involved in age-related declines [194,195,196,197]. For example, Ng et al. [195] studied the functionality of thyroid hormone receptor (TR) β isoforms in relation to the aging cochlea using C57BL/6J mice. They found that TR β1-deficient mice had elevated thresholds compared to control mice; thus, showing that the β1 isoform is involved in the maintenance of cochlear HCs’ survival and auditory function. Human studies have shown that treatment with thyroid hormone, specifically L-thyroxine, could help hypothyroid patients with hearing loss [198]. Of the 30 subjects in the study, nearly half reported improved hearing abilities after 6 months of treatment and 15% restored normal hearing. Another hormone, insulin/insulin-like growth factor (IGF) has been linked to various types of hearing loss, including ARHL [199,200,201,202]. One clinical study performed by Attias et al. [203] reported that IGF-1 hormone treatment on 11 human subjects with Laron syndrome showed no signs of hearing loss and no auditory hypersensitivity. Animal studies have shown parallel results, where IGF treatment was successful in protecting cochlear structures, such as HCs, and reducing ABR thresholds for noise exposure and neomycin ototoxicity [199,201,202].

**Figure 5:**
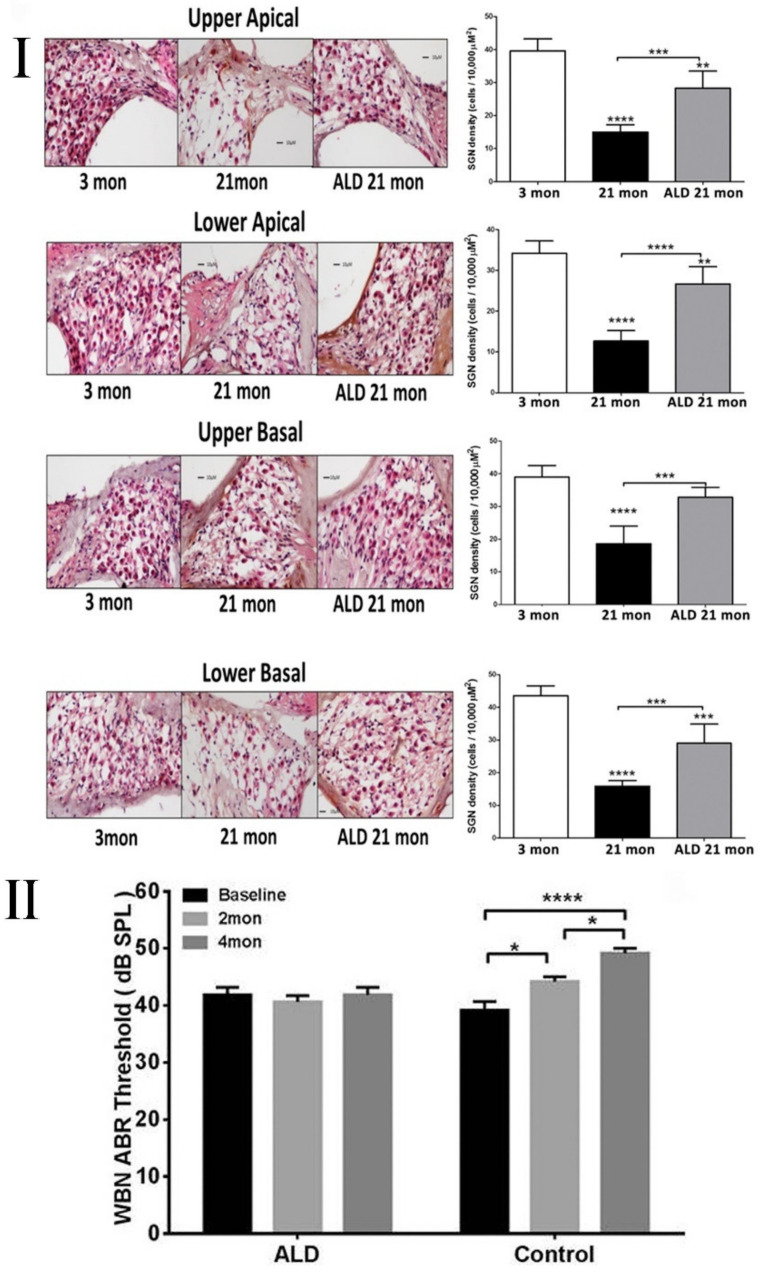
Protective effects of aldosterone treatment on SGN and ABR thresholds. (**I**) Spiral ganglion neuron (SGN) counts between young adult mice (left panel), middle-aged mice without aldosterone treatment (21 months, middle panel), and middle-aged mice with aldosterone treatment (21 months, right panel) show treated animals having higher SGN densities compared to nontreated animals. Magnification: 20 × 1.6. The bar graphs on the right show the quantified SGN cell density with mean ± S.E.M. for each subject group (**II**). Wideband noise mean ABR thresholds of CBA/CaJ mice measured before aldosterone treatment and at 2 and 4 months following treatments show control mice have increased thresholds consistent with ARHL, while aldosterone-treated mice had more stable thresholds. * *p* < 0.05, ** *p* < 0.01, *** *p* < 0.001, **** *p* < 0.0001. From Frisina et al. [113] and Halonen et al. [111], with permission from the publishers.

Aldosterone is another potential candidate for the prevention/treatment of hearing loss involving inflammatory processes [38,111,113,204,205,206]. CBA/CaJ mice (15–18 months) that underwent aldosterone treatment for four months displayed stable ABR thresholds, while the control mice showed an increase in their thresholds, characteristic of ARHL, as shown in Figure 5 [111]. Moreover, the ABR peak 1 and 4 amplitudes for the treated mice were also stable during the treatment period, while control mice displayed decreased amplitudes, suggesting that this hormone afforded some protection from synaptopathy. Aldosterone was also found to regulate protein expression and the activity of NKCC1 channels rapidly and with high sensitivity [204,205]. Following aldosterone treatment, the downregulation of NKCC1 channels in the lateral wall of the cochlea was inhibited. Furthermore, it was found that the survival rate of spiral ganglion neurons and HCs was significantly higher following aldosterone treatment due to the prevention of the downregulation of aldosterone mineralocorticoid receptors [113,204,205]. Improved mineralocorticoid receptor (MCR) expression by aldosterone-treated mice revealed a link to the inhibition of apoptosis previously found in the aging cochlea.

Another group of naturally occurring hormones that have been hypothesized to be therapeutic are estrogen and progesterone/progestin. An animal study conducted by Williamson et al. [207] randomly divided 70 CBA/CaJ mice into 4 groups: placebo, estrogen treated, progesterone treated, and estrogen + progesterone treated. These animals underwent hormone replacement therapy (HRT) for 6 months. The estrogen-only animal group showed better hearing than the other groups, including the control mice who displayed increasing ABR threshold readings consistent with ARHL. The progesterone group showed better hearing than the combination group, which had the worst hearing of all groups. The combination treatment group displayed accelerated ARHL; thus, they had higher ABR thresholds and lower DPOAE amplitudes compared to all other groups, including the control mice. These HRT effects remained even after the treatments were stopped.

The reasoning behind why the combination group showed the worst hearing is still under investigation. A clinical study conducted by Guimaraes et al. [208] displayed similar results. In their study of 124 postmenopausal women divided into similar groups as the Williamson and coworkers [207] study—estrogen alone, estrogen + progestin, and control—they found that the combination group had poorer hearing abilities compared to the estrogen-alone and control groups. The combination group displayed higher pure-tone thresholds, lower DPOAE amplitudes, and poorer speech perception in background noise. The estrogen-alone group had better DPOAE amplitudes and speech perception than the combination group; however, it was worse than the control group. While this study suggests that HRT is not effective in preserving hearing, it shows promising results with estrogen alone in the animal studies.

Mitochondrial intervention strategy is a relatively new therapeutic option that has been gaining more attention recently. Mitochondria play a critical role in cochlear function and dysfunction and seeking out a way to regulate mitochondrial processing is critical for maintaining homeostasis in the inner ear [209,210]. A method to intervene and treat oxidative stress is through the use of antioxidants. A polyunsaturated phosphatidylcholine (PPC), known as lecithin, conserves antioxidant enzymes within the mitochondrial membrane that protects cellular functioning from damage by ROS [211]. In the study by Seidman et al. [211], lecithin was administered to a group of aging rats (18–20 months) for six months. The treated group of rats showed significantly preserved ABR hearing thresholds compared to the control non-treatment group. Moreover, they found the treated animals had elevated membrane potentials and a decline in aging mitochondrial DNA (mtDNA) elimination [211]. Le and Keithley [212] conducted a study where they fed dogs a high-antioxidant diet over a 3-year period. This diet resulted in decreased cellular degeneration of spiral ganglion cells and SV at the base and apex of the cochlea, restoring levels found in young adult dogs, and the opposite effects for the control, non-diet group. Someya et al. [213] orally administered 17 antioxidants (acetyl-l-carnitine, α-lipoic acid, β-carotene, carnosine, coenzyme Q_10_, curcumin, d-α-tocopherol, egcg, gallic acid, lutein, lycopene, melatonin, n-acetyl-l-cysteine, proanthocyanidin, quercetin, tannic acid, resveratrol) to C57BL/6J mice for 11 months and found that α-lipoic acid was successful at almost fully preserving the hearing of the mice. Both α-lipoic acid and coenzyme Q_10_ suppressed Bak, a mitochondrial pro-apoptotic gene, expression in the cochlea; thus, reducing cochlear cell death and preventing some aspects of ARHL. However, there have also been studies that have shown no effects for antioxidant treatments. An example of this was a study carried out by Seidman [214], who examined the effects of vitamin E, vitamin C, melatonin, lazaroid, and 30% calorie restriction, on the hearing of aging 344 Fisher rats. The only group that showed results better than the control group was the calorie-restricted group. Human studies with antioxidant treatment also showed mixed results. The Takumida and Anniko group conducted a study that showed positive results following antioxidant treatment. They gave people 70 years old and above rebamipide and α-lipoic acid, and significant hearing improvement was seen [215,216]. However, a study with 120 patients aged 60 and older were given either ginkgo biloba dry extract, α-lipoic acid combined with vitamin C, papverine chlorhydrate combined with vitamin E, or a placebo, and there were no significant differences between the experimental and control groups [217].

## 7. Conclusions

Inflammaging affects multiple areas and functions within the inner ear, including ion channels, transporters, and mitochondrial functionality. Inflammaging could be a biomechanism for age-related cochlear dysfunction, impacting cellular integrity by modulating ion transport and mitochondrial production. Some of the mitochondrial characteristics and ion channels/transporters discussed above are relatively well established in immune and inflammasome activation in other systems, which may be associated with cochlear aging processes; hence, the involvement of other ion channels and more detailed mitochondrial reaction(s) in response to inflammation still needs to be investigated in more detail. Furthermore, a clearer knowledge of the ionic conductances in mitochondria related to age-triggered inflammation in the inner ear (cochlea) is necessary for us to understand the key mechanisms involved in alteration of ionic homeostasis and mitochondrial dysregulation. Discovering these inner ear biological mechanisms and processes in response to age-related inflammatory changes will further provide new targets for biotherapeutics, which could be used to prevent or treat many aging-associated inflammatory disorders, including ARHL.

## Data Availability

Not applicable.
